# Supplementary material for: The Impact of Heatwaves on Mortality and Morbidity and the Associated Vulnerability Factors: A Systematic Review
Source: Int J Environ Res Public Health. 2022 Dec 6;19(23):16356. doi: 10.3390/ijerph192316356 (PMC9738283; doi:10.3390/ijerph192316356)
Supplement: Supplementary file 1 [file ijerph-19-16356-s001.zip › ijerph-2011648-supplementary.pdf]

Table S1. Keywords search used in the screening process.

Database	Search String
Scopus	TITLE-ABS-KEY ((heatwave* OR heat*) AND (vulnerable* OR sensitivity OR susceptible* OR exposed OR disadvantage* OR risk*) AND (mortality OR death* OR fatal* OR lethal* OR morbidity OR “heat-related illness*” OR “hospital admission*” OR “heat stress*” OR “cardiovascular*” OR “respiratory*” OR asthma OR “chronic obstructive pulmonary disease*” OR “renal*” OR “mental*” OR “ambulance*” OR “emergency department”))
Web of Science	TS=((heatwave* OR heat*) AND (vulnerable* OR sensitivity OR susceptible* OR exposed OR disadvantage* OR risk*) AND (mortality OR death* OR fatal* OR lethal* OR morbidity OR “heat-related illness*” OR “hospital admission*” OR “heat stress*” OR “cardiovascular*” OR “respiratory*” OR asthma OR “chronic obstructive pulmonary disease*” OR “renal*” OR “mental*” OR “ambulance*” OR “emergency department”))
EBSCOhost	((heatwave* OR heat*) AND (vulnerable* OR sensitivity OR susceptible* OR exposed OR disadvantage* OR risk*) AND (mortality OR death* OR fatal* OR lethal* OR morbidity OR “heat-related illness*” OR “hospital admission*” OR “heat stress*” OR “cardiovascular*” OR “respiratory*” OR asthma OR “chronic obstructive pulmonary disease*” OR “renal*” OR “mental*” OR “ambulance*” OR “emergency department”))
PubMed	((heatwave* OR heat*) AND (vulnerable* OR sensitivity OR susceptible* OR exposed OR disadvantage* OR risk*) AND (mortality OR death* OR fatal* OR lethal* OR morbidity OR “heat-related illness*” OR “hospital admission*” OR “heat stress*” OR “cardiovascular*” OR “respiratory*” OR asthma OR “chronic obstructive pulmonary disease*” OR “renal*” OR “mental*” OR “ambulance*” OR “emergency department”))

Table S2. Navigation Guide Systematic Review Ratings of Each Study.

No	Study	Recruitment	Blinding	Exposure Assessment	Outcome Assessment	Confounding	Incomplete Outcome Data	Selective Reporting	Conflicts of Interest	Other Bias	Overall Risk of Bias
1.	Lin et al. 2012	Low	Low	Low	Low	Low	Low	Low	Low	Low	Low
2.	Ahmadnezhad et al. 2013	Low	Low	Low	Low	Low	Low	Low	Low	Low	Low
3.	Toloo et al. 2014	Low	Low	Low	Low	Low	Probably Low	Low	Low	Low	Low
4.	Wang et al. 2015	Low	Low	Probably Low	Low	Low	Low	Low	Low	Low	Low
5.	Tong et al. 2015	Low	Low	Low	Low	Low	Low	Low	Low	Low	Low
6.	Green et al. 2016	Probably Low	Low	Probably Low	Low	Probably Low	Probably Low	Low	Low	Low	Probably Low
7.	Soneja et al. 2016	Low	Low	Low	Low	Low	Low	Probably Low	Low	Low	Low
8.	Kang et al. 2016	Low	Low	Low	Low	Low	Low	Low	Low	Low	Low
9.	Phung et al. 2017	Low	Low	Low	Low	Low	Low	Low	Low	Low	Low
10.	Xu et al. 2017	Low	Low	Low	Low	Probably Low	Low	Low	Low	Low	Low
11.	Li et al. 2017	Probably Low	Low	Low	Low	Probably Low	Low	Low	Low	Low	Low
12.	Borg et al. 2018	Low	Low	Probably Low	Low	Probably Low	Low	Low	Low	Low	Low
13.	Cheng et al. 2018	Low	Low	Low	Low	Probably Low	Low	Low	Low	Low	Low
14.	Yin et al. 2018	Low	Low	High	Low	Probably Low	Probably Low	Low	Low	Low	Low
15.	Huang et al. 2018	Low	Low	Low	Low	Low	Low	Low	Low	Low	Low
16.	Zhang et al. 2018	Low	Low	Probably Low	Low	Probably Low	Probably Low	Low	Low	Low	Probably Low
17.	Campbell et al. 2019	Low	Low	Low	Low	Low	Probably Low	Low	Low	Low	Low
18.	Li et al. 2019	Low	Low	Low	Low	Low	Probably Low	Probably Low	Low	Low	Low
19.	Xu et al. 2019	Low	Low	Low	Low	Probably Low	Probably Low	Probably Low	Low	Low	Probably Low
20.	Patel et al. 2019	Low	Low	Low	Low	Low	Low	Low	Low	Low	Low
21.	Zhao et al. 2019	Low	Low	Low	Low	Low	Probably Low	Low	Low	Low	Low
22.	Xu et al. 2019	Low	Low	Low	Low	Low	Low	Low	Low	Low	Low
23.	Liss et al. 2019	Low	Low	Probably Low	Low	Probably Low	Probably Low	Low	Low	Low	Probably Low
24.	Patel et al. 2019	Low	Low	Low	Low	Low	Low	Low	Low	Low	Low

25.	Kim et al. 2020	Low	Low	Probably Low	Low	Probably Low	Low	Low	Low	Low	Low
26.	Kang et al. 2020	Low	Low	Probably Low	Low	Low	Low	Low	Low	Low	Low
27.	Sohail et al. 2020	Low	Low	Low	Low	Probably Low	Probably Low	Low	Low	Low	Low
28.	Campbell et al. 2021	Low	Low	Low	Low	Probably Low	Probably Low	Low	Low	Low	Low
29.	Kollanus et al. 2021	Low	Low	Probably Low	Low	Probably Low	Low	Low	Low	Low	Low
30.	Wondmagegn et al. 2021	Low	Low	Probably Low	Low	Probably Low	Low	Low	Low	Low	Low
31.	Thompson et al. 2022	Low	Low	Low	Low	Probably Low	Low	Low	Low	Low	Low
32.	Graczyk et al. 2022	Low	Low	Low	Low	Probably Low	Low	Low	Low	Low	Low

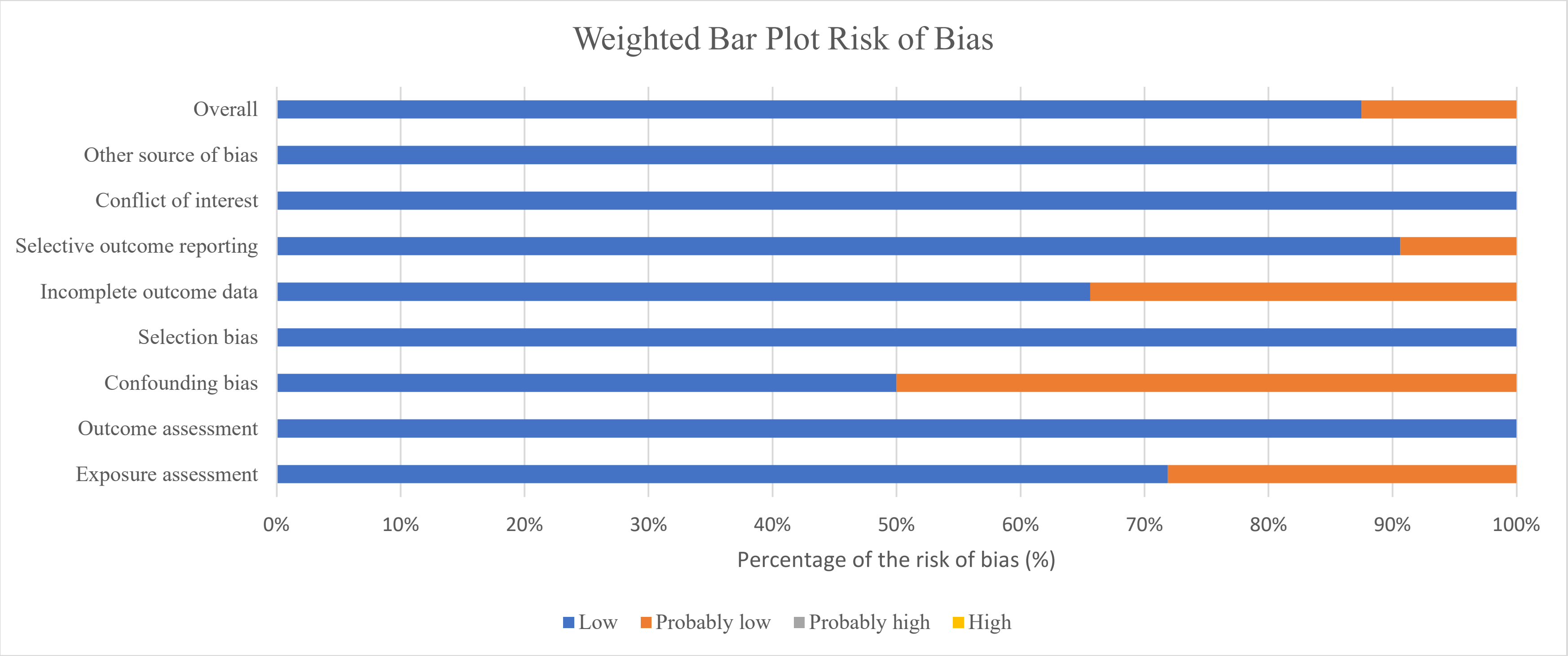

Figure S1. Weighted bar plots indicating percentage of the risk of bias judgments within each bias domain across reviewed studies (n = 32).
